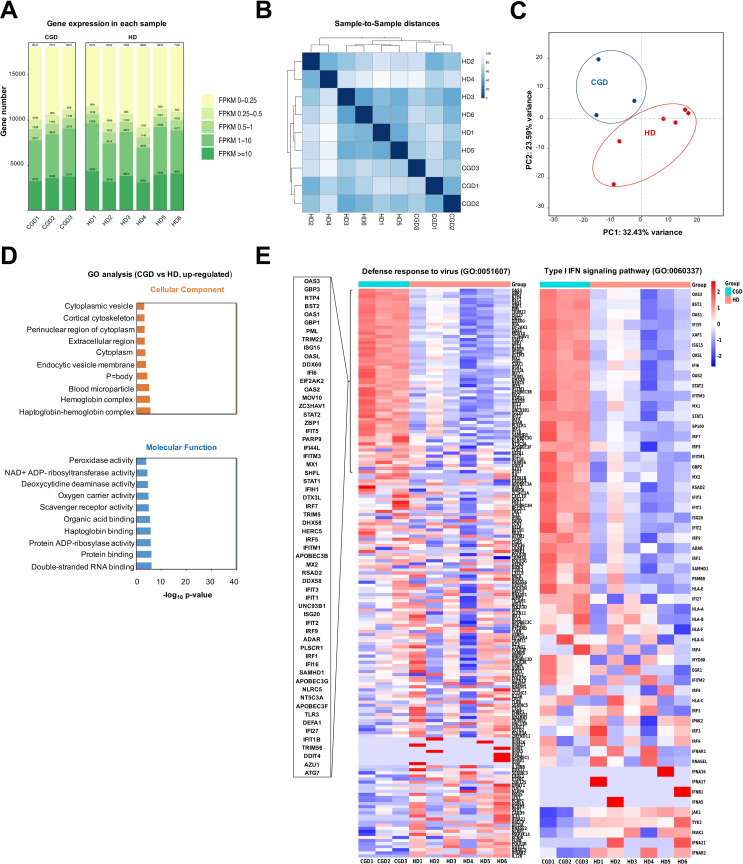# Spontaneous NETosis and type I IFN signaling activation in resting neutrophils of chronic granulomatous disease patients with *CYBB* mutations

**DOI:** 10.1016/j.gendis.2023.101118

**Published:** 2023-09-20

**Authors:** Zhengjing Lu, Wenjun Mou, Jianxin He, Xuedong Ge, Xiaolin Wang, Jingang Gui

**Affiliations:** a Laboratory of Tumor Immunology, Key Laboratory of Major Diseases in Children, Ministry of Education, Beijing Pediatric Research Institute, Beijing Children's Hospital, Capital Medical University, National Center for Children's Health, Beijing 100045, China; b Department of Respiratory Medicine, Beijing Children's Hospital, Capital Medical University, National Center for Children's Health, China National Clinical Research Center for Respiratory Diseases, Beijing 100045, China; c Information Center, PLA Rocket Force Characteristic Medical Center, Beijing 100088, China

## Abbreviations

CGD: chronic granulomatous disease
HD: healthy donors
NADPH: nicotinamide adenine dinucleotide phosphate
ROS: reactive oxygen species
SLE: systemic lupus erythematosus
CYBB: cytochrome b_558_ beta chain
NET: Neutrophil extracellular trap
IFN: interferon
ISGs: interferon-stimulated genes
HBSS: Hank's Balanced Salt solution
PMA: Phorbol 12-myristate 13-acetate
citH3: citrullinated histone H3
DAPI: 4ʹ,6-diamidino-2-phenylindole
DEGs: differentially expressed genes
GO: Gene Ontology
FPKM: fragments per kilobase of exon model per million mapped fragments
PCA: principal component analysis
BP: biological process
CC: cellular component
MF: molecular function
GSEA: gene set enrichment analysis
OAS1: 2′-5′-Oligoadenylate Synthetase 1
OAS2: 2′-5′-Oligoadenylate Synthetase 2
OAS3: 2′-5′-Oligoadenylate Synthetase 3
OASL: 2′-5′-Oligoadenylate Synthetase Like
IFI6: interferon alpha inducible protein 6
IFI27: interferon alpha inducible protein 27
IFIT1: interferon-induced protein with tetratricopeptide repeats 1
IFIT3: interferon-induced protein with tetratricopeptide repeats 3
IFITM3: interferon-induced transmembrane protein 3
IFI44: interferon-induced protein 44
IFI44L: interferon-induced protein 44 like
MX1: MX dynamin like GTPase 1
MX2: MX dynamin like GTPase 2
ISG15: ISG15 ubiquitin like modifier
ISG20: ISG20 ubiquitin like modifier
RSAD2: radical S-adenosyl methionine domain containing 2
BST2: bone marrow stromal cell antigen 2
STAT1: signal transducer and activator of transcription 1
STAT2: signal transducer and activator of transcription 2
IRF7: interferon regulatory factor 7

Mutations in *CYBB*, encoding gp91^phox^ subunit of NADPH oxidase in phagocytes, impair the respiratory burst of neutrophils and result in X-linked chronic granulomatous disease (CGD). While inflammatory response and NETosis are important modalities employed by neutrophils for pathogen clearance, variants in these cell functions in CGD neutrophils (CGD-PMN) could possibly explain the insufficient defense and accumulation of phagocytes in the sites of infection. To decipher the intrinsic features of CGD-PMN, neutrophils from X-linked CGD patients with *CYBB* mutations and age-matched healthy donors (HD-PMN) were compared. Our study found an enhanced spontaneous neutrophil extracellular trap (NET) formation with histone hypercitrullination in resting CGD-PMN. RNA sequencing (RNA-seq) analysis and qPCR validation further revealed a prominent activated type I interferon (IFN) gene signature. This suggested that in CGD patients an inefficient clearance of pathogen caused a chronic stimulation to provoke a spontaneous type I IFN-mediated neutrophil activation and NET formation which were never seen in resting neutrophils of healthy controls.

To understand the effects of the genetic mutation on enzyme expression and neutrophil function in CGD, ten unrelated patients with *CYBB* mutations were enrolled, and the clinical information and laboratory genetic analysis were collected (Table S1). In this study, due to the rarity and severity of the disease, the limitations of clinical practice prevented us from collecting enough blood samples from one patient for all experimental purposes. Thus, samples used for RNA-seq and protein expression detection did not come from the same patients. As one of the main subunits of flavocytochrome b_558_ which is the transmembrane component of NADPH oxidase, gp91^phox^ was detected at mRNA and protein levels in HD-PMN but not in CGD-PMN (Fig. 1A–D). As a validation in other phagocytes, missing of gp91^phox^ expression was also observed in monocytes (Fig. S1). As expected, CGD-PMN without gp91^phox^ exhibited poor ROS production upon PMA stimulation (Fig. 1E, F), which was in accordance with CGD patients previously reported.^1^

**Figure 1:**
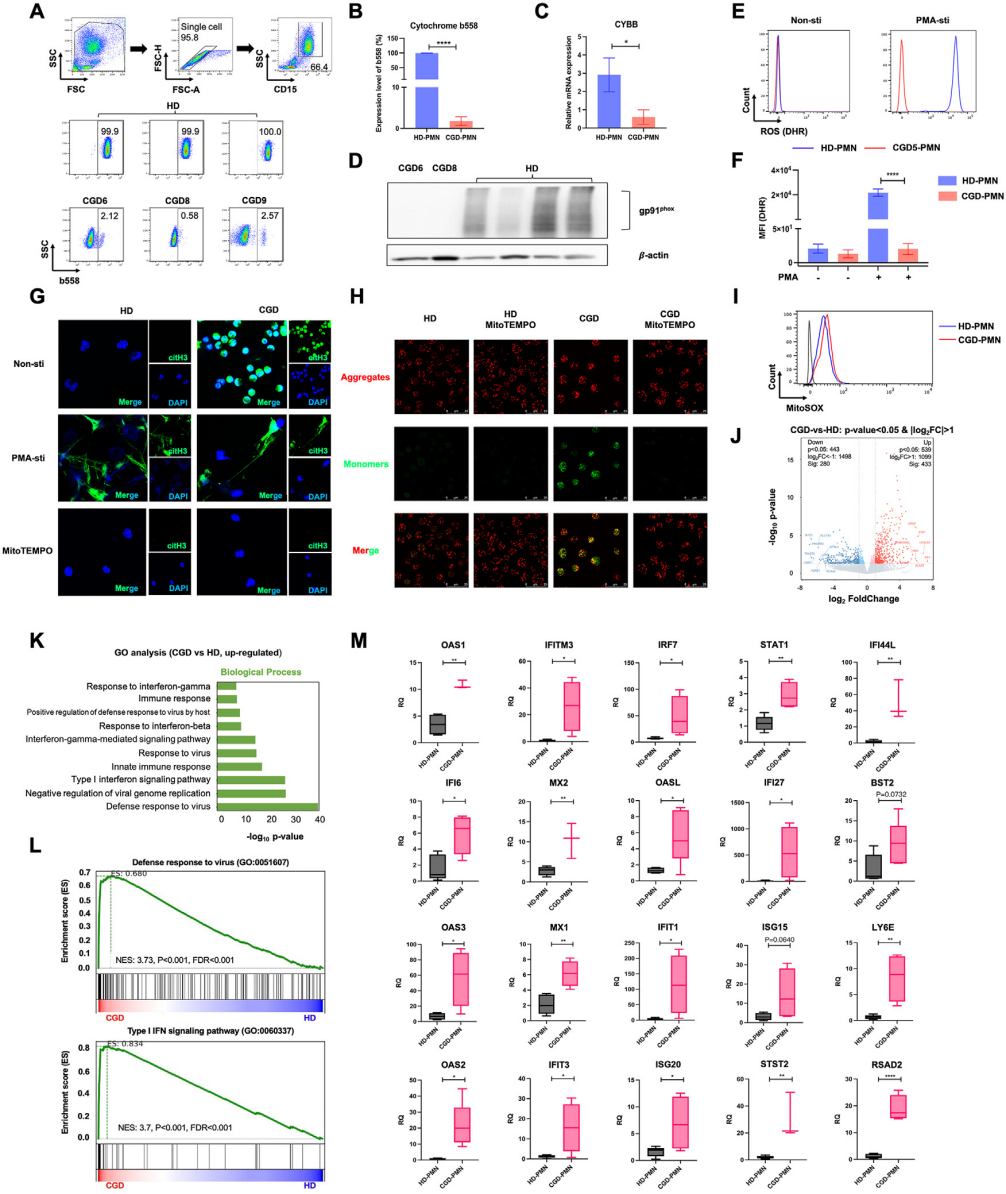
Functional analysis and gene expression profile of CGD-PMN. **(A, B)** Flow cytometry dot plots and positive proportions of cytochrome b_558_ expression in neutrophils (CD15) from CGD patients and healthy donors. **(C)** Relative mRNA level of *CYBB* expression was detected by qPCR with GAPDH as the endogenous control. **(D)** gp91^phox^ expression in CGD-PMN and HD-PMN was detected by Western blot. **(E)** FACS histograms for ROS generation in CGD5-PMN and HD-PMN before and after stimulation with PMA (100 nM). **(F)** Mean fluorescence intensity (MFI) values of DHR123 were shown by bar diagrams. **(G)** Confocal images of NET formation in non-stimulated and PMA-stimulated neutrophils, and non-stimulated neutrophils with 100 μM MitoTEMPO. Citrullinated histone H3 (citH3) was in green and DAPI was in blue. Original magnification = 63 × . **(H)** HD-PMN and CGD-PMN were seeded into 35-mm glass-bottom dished and stained with JC-1 (JC-1 aggregates: red; JC-1 monomers: green). Scale bars = 100 μm. **(I)** The histograms showing MitoSOX Red-based flow cytometric detection of mitochondrial ROS production. **(J)** The volcano plot of differential gene expression between CGD-PMN and HD-PMN. **(K)** GO analysis for the potential pathways of up-regulated DEGs. **(L)** Gene set enrichment analysis for the potential function of all DEGs. **(M)** qPCR results for type I IFN-related gene expression in CGD-PMN, normalized to GAPDH expression. The results for all measurements were displayed as mean ± SEM. *P* value > 0.05 indicated no statistically significant difference (ns) (^∗^*P* < 0.05, ^∗∗^*P* < 0.01, ^∗∗∗∗^*P* < 0.0001).

To our surprise, spontaneous NETosis was found in resting CGD-PMN but not HD-PMN (Fig. 1G). Immunofluorescence staining revealed that histone-hypercitrullinated NETs characteristic of polymorphonuclear appearance with intact nuclear membranes. By contrast, upon PMA stimulation, both CGD-PMN and HD-PMN initialized strong NETosis, which appeared to rupture and release long stretches of extensively decondensed chromatin into the extracellular space, forming web-like chromatin structures. It has been known that the generation of ROS is a prerequisite for NETosis. Now that patients have a defect ROS production due to *CYBB* mutation, a spontaneous NET formation must have been induced through other ROS resources. Accumulating evidence revealed that mitochondrion is another major site of ROS generation, and mitochondrial ROS synthesis could be sufficient to generate NETs in systemic lupus erythematosus and CGD.^2^ Indeed, through MitoSOX Red staining, a specific dye for mitochondrial ROS, we proved that mitochondrial superoxide production was higher in CGD-PMN (Fig. 1I). Different from typical suicidal NETosis, our confocal microscope further revealed that the spontaneous NETs in CGD-PMN were phenotypically prone to vital pathway in which integrity of cell membrane was preserved to allow the release of NETs via vesicular exportation. Immunofluorescence results using JC-1 indicated that CGD-PMN had a decreased mitochondrial membrane potential (Fig. 1H). The NET formation was abrogated after treating CGD-PMN with MitoTEMPO, a mitochondria-specific superoxide scavenger, suggesting that spontaneous NETosis in CGD-PMN relied on mitochondrial ROS production (Fig. 1G). The dominant mitochondrial ROS production-induced vital NETosis in CGD-PMN might account for delayed pathogen clearance and accumulated neutrophils in the inflammatory site leading to tissue granulomatosis.^3^ In sum, our study demonstrated the dependence of mitochondrial ROS synthesis and its effects on inducing spontaneous NETs in the absence of NADPH oxidase activity in CGD patients.

To explore if there are differences between HD-PMN and CGD-PMN at the molecular level, HD-PMN (*n* = 6) and CGD-PMN (*n* = 3) were isolated and subjected to high-throughput RNA-seq analysis. We found that the general gene expression distribution of the total fragments per kilobase of exon model per million mapped fragments (FPKM) values showed similarity, whereas the individual FPKM values were different amongst the 6 HD-PMN and 3 CGD-PMN (Fig. S2A). As expected, the indicated 6 HD-PMN or 3 CGD-PMN respectively manifested closer clustering characteristics in gene expression pattern as confirmed by the heatmap diagram (Fig. S2B) and principal component analysis (Fig. S2C). The distinguishable landscape of gene expression pattern between CGD-PMN and HD-PMN revealed a total number of 433 up-regulated and 280 down-regulated genes (*P* < 0.05, |log_2_FC| ≧ 1) (Fig. 1J). Gene Ontology (GO) analysis for biological process (BP) using the screened differentially expressed genes (DEGs) revealed that immunologically relevant signaling pathways, including defense response to the virus and response to IFN, were enriched in CGD-PMN (Fig. 1K; Fig. S2D). In accordance with GO analysis, gene set enrichment analysis (GSEA) also indicated that pathways including defense response to the virus (GO: 0051607) and type I IFN signaling pathway (GO: 0060337) were collectively enriched in the CGD-PMN (Fig. 1L). The expression levels of DEGs involved in these two pathways were illustrated in hierarchical clustering heatmaps (Fig. S2E). Genes of enhanced expression in both virus defense and type I IFN pathways include OAS1, OAS2, OAS3, OASL, IFI6, IFI27, IFITM3, IFI44L, MX1, MX2, ISG20, STAT1, STAT2, RSAD2, ISG15, and BST2. Most of them are active participants in IFN signaling and inflammatory responses, which agrees with the pro-inflammatory manifestation in CGD patients. The up-regulation of the above interferon-stimulated genes (ISGs) in CGD-PMN was validated by qPCR and reassured our belief that an active type I IFN gene signature existed in resting CGD-PMN (Fig. 1M; Table S2). Active type I IFN signaling is responsible for the recruitment of neutrophils to sites of infection, regulation of neutrophil function, and immunopathogenesis.^4^ In fact, CGD was previously identified as a type I IFN autoimmune disease manifested by up-regulated ISGs in peripheral blood after stimulation.^5^ In our study, resting CGD-PMN was shown to have increased ISG expression which suggested a pro-inflammatory status presented as a consequence of chronic stimulation due to insufficient phagocyte ROS production. In addition, elevated mitochondria-derived ROS in resting CGD-PMN could be a cause or a consequence of ISG up-regulation. This observation agreed with the previous report that a mitochondrial ROS-dependent NETosis of low-density granulocytes from individuals with CGD can promote externalization of pro-inflammatory oxidized mtDNA and subsequent activation of STING and/or TLR-dependent type I IFN synthesis.^2^ Our study proved an elevated mitochondrial ROS production accompanied by a type I IFN signaling activation in resting neutrophils in CGD patients in the absence of NADPH oxidase activity. However, the key drivers of spontaneous NETosis and the role of type I IFNs underlying the NETotic phenotype of patient cells require further exploration.

The main limitation of the current study is that there are few CGD patients for whom enough neutrophils are available for research purposes, and cell number from one patient is limited, which restricted us from applying them to all the experiments (flowcytometry, immunofluorescence, Western blot, RNA-seq, qPCR, *etc*.). Besides, we cannot collect enough CGD samples to refine our data. As different patient samples were obtained at various disease stages, it is hard to know how the chronic stimulation time and the pathogen the patient encountered shape the neutrophil function.

In summary, distinguished from HD-PMN, impaired functional gp91^phox^ expression and extremely poor ROS production, but enhanced spontaneous NET formation with histone hypercitrullination in resting CGD-PMN were observed in our study. Sustained type I IFN signaling was associated with resting CGD-PMN and could contribute to the pro-inflammatory manifestation in CGD patients. However, whether these features resting CGD-PMN possessed have anything to do with inefficient pathogen clearance remains to be further explored experimentally.

### Conflict of interests

The authors declare that there is no conflict of interests.

### Funding

This work was supported by grants from the 10.13039/501100001809National Natural Science Foundation of China (No. 82173084, 82002637).

## Figures and Tables

**figs1:**
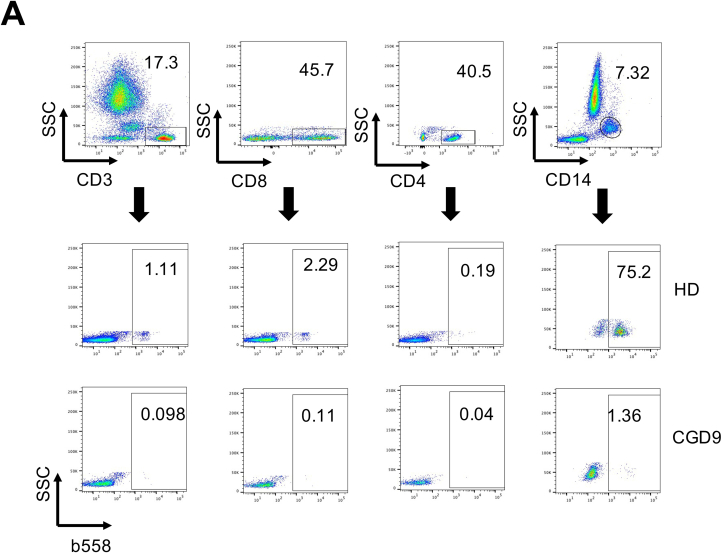


**figs2:**