# Supplementary material for: Spontaneous NETosis and type I IFN signaling activation in resting neutrophils of chronic granulomatous disease patients with CYBB mutations
Source: Genes Dis. 2023 Sep 20;11(6):101118. doi: 10.1016/j.gendis.2023.101118 (PMC11278796; doi:10.1016/j.gendis.2023.101118)
Supplement: Multimedia component 1 [file mmc1.docx]

**Supplementary material**

**Methods**

**Patient samples and genetic analysis**

Patients were referred to the Beijing Children's Hospital Affiliated to the Capital Medical University. The study was approved by the Medical Ethics Committee of Beijing Children’s Hospital, the Capital Medical University. Research purpose and informed consent were signed by the parents/guardians of all the patients.

CGD was diagnosed based on clinical features and severe recurrent infections, and was confirmed by functional, molecular and genetic analyses. Clinical manifestations and laboratory findings were collected from electric medical records. Blood was collected from ten children with CGD and ten age-matched voluntary healthy donors for physical examination. Genomic DNA was extracted from peripheral blood cells using mini blood kit (QIAGEN, Valencia, CA, USA), following the manufacturer instructions. Direct DNA sequencing of all exons and exon-intron boundaries of the *CYBB* gene was performed as described in a previous publication [1]. The sequenced data were further analyzed by the NCBI database (<http://www.ncbi.nlm.nih.gov/BLAST>) and the Ensembl SNPs database (<http://www.ensembl.org>).

**Neutrophil isolation**

Human circulating neutrophils were isolated from heparin-anticoagulated blood of CGD patients and healthy donors by dextran sedimentation followed by density gradient centrifugation according to the lymphocyte separation solution (TBD science, Tianjin, China). To remove contaminating erythrocytes, the cell suspension was exposed to red cell lysis buffer for 5 min and then washed with Hank's Balanced Salt solution (HBSS) (Gibco, Invitrogen, Carlsbad, CA, USA). Gently, this step was performed twice with cell pellets by centrifugation at 1600 rpm for 5 min. The purity of the PMN preparations and cell viability were routinely assessed by flow cytometry (LSRFortessa X-20, BD Biosciences, San Jose, CA, USA) and neutrophil proportions were more than 95%.

**Detection of ROS levels**

The total ROS detection probe (Dihydrorhodamine 123, DHR123, Sigma Aldrich, Saint Louis, MO, USA) and mtSOX Deep Red (Dojindo, Kumamoto, Japan) were used according to the manufacturer’s instructions [2]. In brief, neutrophils were incubated with DHR123 or mtSOX and cells were immediately analyzed by measuring fluorescence using BD LSRFortessa X-20 flow cytometry (BD Biosciences, San Jose, CA, USA).

**Western blot**

Expression of gp91^phox^ in CGD-PMNs was evaluated by western blot as described [3]. In brief, 40 $\mu$g protein extracted from each sample was separated on 10% SDS-PAGE, transferred to a polyvinylidene fluoride membrane, and blotted with a rabbit anti-gp91^phox^ monoclonal antibody (Santa Cruz Biotechnology, Santa Cruz, CA, USA) at 4 °C for 16 h. After that, the membrane was incubated with goat anti-rabbit IgG (H+L) HRP-conjugated secondary antibody (OriGene Technologies, Rockville, USA). GAPDH (Proteintech, Rosemont, IL, USA) was used as a loading control. The antigen-antibody complexes were visualized by enhanced chemiluminescence (ECL, Merck Millipore, Billerica, MA, USA).

**Flow cytometry analyses of gp91^phox^ protein expression**

Expression of gp91^phox^ on erythrocytes-removed peripheral blood cells were detected by flow cytometry using FITC-labeled anti-flavocytochrome b_558_ monoclonal antibody (7D5 mAb, Marine Biological Laboratory, Woods Hole, MA, USA). For surface staining of lymphocytes, monocytes and neutrophils from whole blood, the following fluorochrome-conjugated monoclonal antibodies were used (Anti-human CD3, CD4, CD8, CD14, CD15) (BioLegend, San Diego, CA, USA) at 4 °C for 30 min in dark. Data were acquired on a BD LSRFortessa X-20 flow cytometer and analyzed using FlowJo software (version 10.8.1; BD Biosciences, San Jose, CA, USA).

**NETosis detection by immunofluorescence staining**

Purified neutrophils were resuspended at 1 × 10^6^ cells/ml RPMI 1640 and 100 μl was added into poly-L-Lysine (Sigma-Aldrich, St. Louis, MO, USA) pre-coated confocal dishes. Following stimulation of 100 nM PMA or treated with 100 µM MitoTEMPO (Sigma Aldrich, Saint Louis, MO, USA) for 2 h at 37 °C, neutrophils were fixed in 4% paraformaldehyde and permeabilized with 0.1% Triton X-100 followed by incubation with blocking buffer (PBS containing 5 mg/mL Fraction V BSA). Cells were stained with rabbit anti-citrullinated histone H3 (citH3) polyclonal antibody (1:100 dilution; Abcam, Cambridge, CB2 0AX, UK) in blocking buffer at 4 °C overnight. Then donkey anti-rabbit IgG H&L secondary antibody conjugated with AlexaFluor 488 (1:400; Abcam, Cambridge, CB2 0AX, UK) in blocking buffer were added and incubated at RT for 2 h in dark. The DNA was stained with 4ʹ,6-diamidino-2-phenylindole (DAPI) for 10 min. NETosis was confirmed by imaging the colocalization of citH3 and DAPI and captured by utilizing a Leica SP8 laser scanning confocal microscopy (Leica Microsystems Inc. Deerfield, IL, United States).

**Assessment of mitochondrial membrane potential**

Neutrophils were incubated with JC-1 (Beyotime, China) at 37 °C for 30 min to evaluate the mitochondrial membrane potential. Cells were washed twice and the fluorescence intensity of the JC-1 monomers/aggregates (green fluorescence for monomer, red fluorescence for aggregate) were taken by Leica SP8 laser scanning confocal microscopy, following the instructions of JC-1 assay kit.

**RNA‐Seq and bioinformatic analysis**

RNA-seq analysis was performed in CGD-PMN and age-matched HD-PMN. Total RNA of neutrophils was extracted using TRIzol reagent (Invitrogen, Carlsbad, CA, USA) and stored at -80 °C for subsequent RNA-seq (OEbiotech, Shanghai, China). Differential expression analysis was performed using DESeq2 R package (1.10.1). Gene expression level with log_2_ fold change ≧ 1 or ≦ -1 and an adjusted *p*-value < 0.05 when comparing CGD-PMN with HD-PMN were defined as DEGs in further analysis. The cut-off value for significant GO and Kyoto Encyclopedia of Genes and Genomes (KEGG) results was *p* < 0.05 and false discovery rate (FDR) < 0.25. The bioinformatic analyses were performed according to the instructions of the databases and online platforms.

**Quantitative PCR (qPCR)**

Total RNA from CGD-PMN was purified using the Direct-zol RNA Miniprep (ZYMO research, Orange, CA, USA) following the manufacturer’s instructions. Total RNA concentrations were measured by a NanoDrop ND-8000 (Thermo Fisher Scientific Inc., Waltham, MA, USA). Reverse transcription was performed according to standard protocols using a RevertAid First Strand cDNA Synthesis Kit (Thermo Fisher Scientific Inc., Waltham, MA, USA). qPCR was performed using SYBR Green PCR Master Mix (TIANGEN, Beijing, China) and the fluorescence was recorded by a QuantStudio 6 flex real-time PCR system (Applied Biosystems, Foster City, CA, USA). Relative expression was calculated by the 2^-ΔΔCt^ method with GAPDH as the endogenous housekeeping gene control. Primer sequences are listed in Table 1.

**Statistical Analysis**

The statistical differences between two groups were analyzed with Student’s t-test by using GraphPad Prism software version 8.0 (GraphPad Software, Inc., San Diego, CA, USA). Significant differences between groups were represented by * *p* < 0.05, ** *p* < 0.01 and **** *p* < 0.0001.

**References**

[1] Al-Zadjali S, Al-Tamemi S, Elnour I, et al. Clinical and molecular findings of chronic granulomatous disease in Oman: family studies. *Clin Genet*. 2015; 87(2):185-189. doi:10.1111/cge.12351.

[2] Vowells SJ, Sekhsaria S, Malech HL, et al. Flow cytometric analysis of the granulocyte respiratory burst: a comparison study of fluorescent probes. *J Immunol Methods*. 1995; 178(1):89-97. doi:10.1016/0022-1759(94)00247-t.

[3] Zhang H, Chai W, Yang W, et al. The increased IL-17-producing gammadeltaT cells promote tumor cell proliferation and migration in neuroblastoma. *Clin Immunol*. 2020; 211(108343. doi:10.1016/j.clim.2020.108343.

**Supplementary Figure Legends**

**Fig. S1 Genotypic characterization of *CYBB* variants**

(**A**) Molecular variants identified in different exons of *CYBB* in current study. Two novel mutations had been highlighted in red. (**B**) Representative electropherograms showed eight different *CYBB* variants. Red arrows indicated the mutation sites.

**Fig. S2 Cytochrome b558 expression**

(**A**) Flow cytometry dot plots of cytochrome b_558_ expression in CD3, CD8, CD4 and monocytes (CD14) from CGD9 and an age-matched healthy donor.

**Fig. S3 CGD-PMN exhibited distinguishable landscape of gene expression profiling**

(**A**) The distributions of gene expression in CGD-PMN (n=3) and HD-PMN (n=6) based on gene number. Different colors in the figure represent different ranges of FPKM values. (**B**) Correlation analysis of CGD-PMN and HD-PMN by heatmap diagram. (**C**) Principal component analysis (PCA) of CGD-PMN and HD-PMN. (**D**) GO analysis for the potential pathways of CGD-PMN up-regulated DEGs, including cellular component, (CC), and molecular function (MF). (**E**) The hierarchical clustering heatmap for defense response to virus-related and Type I IFN signaling pathway-related genes in CGD-PMN and HD-PMN.
