# Supplementary material for: Spontaneous NETosis and type I IFN signaling activation in resting neutrophils of chronic granulomatous disease patients with CYBB mutations
Source: Genes Dis. 2023 Sep 20;11(6):101118. doi: 10.1016/j.gendis.2023.101118 (PMC11278796; doi:10.1016/j.gendis.2023.101118)
Supplement: Multimedia component 2 [file mmc2.docx]

**Table S1 Clinical characteristics and genotypical profiles of CGD patients**

| **Patient ID** | **Age at symptom onset** **(months)** | **Age at diagnosis (months)** | **Gender** | **Pathogens** | **Clinical manifestations** | **CRP (<8mg/L)** | **cDNA nucleotide change** | **Type of mutation** | **Gene location** |
| --- | --- | --- | --- | --- | --- | --- | --- | --- | --- |
| CGD1 | 54 | 56 | M | *Aspergillus flavus, Aspergillus oryzae* | Recurrent fever and severe pneumonia | 13 | c.252G>A | Splice site | Exon 3 |
| CGD2 | 13 | 15 | M | *Candida albicans, rotavirus, Stenotrophomonas maltophilia, lautropia mirabilis* | Prolonged fever, sepsis, rotavirus enteritis, pneumonia and perianal abscess | 20 | c.469C>T | Nonsense | Exon 5 |
| CGD3 | 1 | 14 | M | *Serratia marcescens*, human herpesvirus 6 (HHV-6) | Sepsis, acute hepatic failure, status epilepticus, viral encephalitis, pneumonia, perianal abscess, osteomyelitis, deep venous thrombosis, hypoproteinemia | 38 | c.676C>T | Nonsense | Exon 7 |
| CGD4 | 1 | 2 | M | *Serratia marcescens* | Severe pneumonia, liver function impairment, conjunctivitis | 54 | c.676C>T | Nonsense | Exon 7 |
| CGD5 | 15 | 17 | M | - | Recurrent fever and severe pneumonia | - | c.1038delT | Deletion | Exon 9 |
| CGD6 | 2 | 14 | M | *Staphylococcus aureus, Aspergillus* | Recurrent perianal abscess, severe pneumonia, left axillary lymph node enlargement, left axillary lymph node tuberculosis, anal fistula, inflammatory bowel disease | 8 | c.416_423delTAGCACTCinsAGAGT | Deletion/ insertion | Exon 5 |
| CGD7 | 1 | 8 | M | *Aspergillus fumigatus, cytomegalovirus, Streptococcus* | Severe pneumonia, tuberculosis infection, perianal abscess, chronic diarrhea | 9 | c.1210-1212delGTG | Deletion | Exon 10 |
| CGD8 | 12 | 12 | M | *Haemophilus influenzae, Staphylococcus, Leckercia adecarboxylata, Burkholderia gladiolus* | Recurrent fever, sepsis, pneumonia, skin infections, tuberculosis infections | 44 | c.898-8_904del  TTCTATAGGTGGTCA | Splice site/  deletion | Intron 8  Exon 9 |
| CGD9 | 5 | 7 | M | *Staphylococcus aureus, Streptococcus hemolyticus* | Recurrent fever, pneumonia, sepsis, liver abscess, purulent lymphadenitis, tuberculosis infection, liver function impairment | 61 | c.1314+1G>T | Splice site | Intron 10 |
| CGD10 | 52 | 58 | M | - | Recurrent fever, pneumonia | 129.8 | c.370delG | Deletion | Exon 5 |

M: male
