# Supplementary material for: Spontaneous NETosis and type I IFN signaling activation in resting neutrophils of chronic granulomatous disease patients with CYBB mutations
Source: Genes Dis. 2023 Sep 20;11(6):101118. doi: 10.1016/j.gendis.2023.101118 (PMC11278796; doi:10.1016/j.gendis.2023.101118)
Supplement: Multimedia component 3 [file mmc3.docx]

**Table S2 Primers for qPCR.**

| Target gene | Forward primer (5’-3’) | Reversed primer (5’-3’) |
| --- | --- | --- |
| CYBB | CAAGATGCGTGGAAACTACC | TTGAGAATGGATGCGAAGG |
| GAPDH | GGAGCCAAAAGGGTCATCACTC | GAGGGGCCATCCACAGTCTTCT |
| IFI6 | GTTCTCACTATATTGTCCAGGCTAGAGT | AGTTTATTCTGTTTTCACATCTAGGTTGTT |
| IFI27 | TCCAAGCTTAAGACGGTGAGG | ATGGGCACAGCCACAACTC |
| IFIT1 | CTCCTTGGGTTCGTCTACAAATTG | AGTCAGCAGCCAGTCTCAG |
| IFIT3 | TCAGAAGTCTAGTCACTTGGGG | ACACCTTCGCCCTTTCATTTC |
| IFI44L | AATCAGACAGAACAGTTAATCCTC | TCAACCATATCTTCAATGCTACC |
| IFITM3 | ACTGTCCAAACCTTCTTCTCTCC | TCGCCAACCATCTTCCTGTC |
| OAS1 | GTGAGCTCCTGGATTCTGCT | AGGGTACTCATGTGTTCCAATGT |
| OAS2 | CTCAGAAGCTGGGTTGGTTTAT | ACCATCTCGTCGATCAGTGTC |
| OAS3 | GAAGGAGTTCGTAGAGAAGGCG | CCCTTGACAGTTTTCAGCACC |
| OASL | CTGATGCAGGAACTGTATAGCAC | CACAGCGTCTAGCACCTCTT |
| ISG15 | GTTCATGAATCTGCGCCTGC | CAGCCTTTATTTCCGGCCCT |
| ISG20 | TGTTCTGGATGCTCTTGTGC | GCACTGAAAGAGGACATGAGC |
| BST2 | GGTGGAGCGACTGAGAAGA | GCGAAAAGCCGAGCAGGA |
| LY6E | GGGAATCTCGTGACATTTGGC | ACACCAACATTGACGCCTTCT |
| RSAD2 | AGGTTCTGCAAAGTAGAGTTGC | GATCAGGCTTCCATTGCTC |
| IRF7 | TTGGCTCCTGAGAGGGCA | CAGCCCAGGCCTTGAAGATA |
| STAT1 | CCATCCTTTGGTACAACATGC | TGCACATGGTGGAGTCAGG |
| STAT2 | GAAGCTGCACTTGGGAGTGA | GGATCCTGGGAAAAGGGCTG |
| MX1 | CAGCACCTGATGGCCTATCA | ACGTCTGGAGCATGAAGAACTG |
| MX2 | AAACTGTTCAGAGCACGATTGAAG | ACCATCTGCTCCATTCTGAACTG |
